# In randomization we trust? There are overlooked problems in experimenting with people in behavioral intervention trials^☆^

**DOI:** 10.1016/j.jclinepi.2013.09.004

**Published:** 2014-03

**Authors:** Jim McCambridge, Kypros Kypri, Diana Elbourne

**Affiliations:** a Department of Social & Environmental Health, Faculty of Public Health and Policy, London School of Hygiene & Tropical Medicine, 15-17 Tavistock Place, London WC1H 9SH, UK; b Centre for Clinical Epidemiology and Biostatistics, School of Medicine and Public Health, University of Newcastle, Newcastle, Australia; c Department of Medical Statistics, Faculty of Epidemiology and Population Health, London School of Hygiene & Tropical Medicine, Keppel Street, London, UK

**Keywords:** Behavior, Trials, Bias, Research participation, Intervention, Hawthorne effect

## Abstract

**Objectives:**

Behavioral intervention trials may be susceptible to poorly understood forms of bias stemming from research participation. This article considers how assessment and other prerandomization research activities may introduce bias that is not fully prevented by randomization.

**Study Design and Setting:**

This is a hypothesis-generating discussion article.

**Results:**

An additivity assumption underlying conventional thinking in trial design and analysis is problematic in behavioral intervention trials. Postrandomization sources of bias are somewhat better known within the clinical epidemiological and trials literatures. Neglect of attention to possible research participation effects means that unintended participant behavior change stemming from artifacts of the research process has unknown potential to bias estimates of behavioral intervention effects.

**Conclusion:**

Studies are needed to evaluate how research participation effects are introduced, and we make suggestions for how research in this area may be taken forward, including how these issues may be addressed in the design and conduct of trials. It is proposed that attention to possible research participation effects can improve the design of trials evaluating behavioral and other interventions and inform the interpretation of existing evidence.

## 1 Introduction

**What is new?**

• An additivity assumption underlying conventional trial design and analysis is problematic in behavioral intervention trials.
• Pre and postrandomization research participation effects may interact with evaluated interventions.
• Randomization does not fully prevent the introduction of bias via these mechanisms.
• New conceptual and empirical work is needed to better understand these problems.
• Research artifacts in other types of trials should also be amenable to control.

Randomized controlled trials (RCTs) are widely accepted as the most rigorous research designs for the evaluation of the effects of interventions. Behavioral intervention trials are studies in which the primary purpose is to evaluate attempts to influence behavior or the consequences of any resultant behavior change. They are important to public health as lifestyle behavioral risk factors contribute strongly to a wide range of health problems [1]. Data from our best behavioral intervention trials may not, however, be as robust as we currently believe, and it has been suggested that research participation may account for more observed change than evaluated interventions [2]. It has long been known that participants may react in unintended ways to being studied and this may lead to change [3]. It is suggested that this entails largely overlooked potential for bias in behavioral intervention trials. Valid inferences about the true effects of behavioral interventions are hampered by our inability to identify and rule out alternative explanations for behavior change. These concerns have much wider relevance as almost all trials and other types of human research depend on the cooperation of their participants, which may be unwittingly influenced by the way studies are conducted.

## 2 Assessment and other aspects of research participation may change behavior

Taking part in trials typically involves both recruitment and baseline assessment activities before randomization, and subsequently exposure to study conditions and assessment at follow-up. Any or all of these research activities may influence participant cognitions, emotions, and behavior. Formally signing a consent form, for example, may lead to or strengthen commitment to behavior change. Questions answered for research assessment purposes may stimulate new thinking about the behavior, which also may be a prelude to action [4,5].

It is difficult to point to any well-established coherent body of literature investigating these issues. There exist, however, somewhat disparate strands of relevant research, and thinking about research, which relate to different parts of the research process being investigated, or have their origins in specific disciplines or research contexts, or are concerned with specific methodological problems in research. For example, assessment reactivity effects in trials of brief alcohol interventions jeopardize the safety of inferences made because although reactivity effects may be small, the effects of the interventions being evaluated are also small [6]. In this field, because assessment is an integral component of the brief interventions being evaluated, research assessments produce contamination in the form of unwitting exposure of the control group to intervention content [7].

There is a plethora of labels and constructs that have been developed to describe and study similar phenomena. For example, within health psychology, assessment reactivity is conceptualized as “mere measurement,” “question-behavior,” or “self-generated validity” effects [4,5,8]. Synthesizing this type of literature is challenging as many findings have been generated incidentally to the main purposes of the research being undertaken. The idea that being assessed itself influences behavior has, however, been established in the literature for approximately one 100 years [3]. The Hawthorne effect, usually taken to mean that monitoring of a behavior for research purposes changes performance of that behavior, is approximately 60 years old [9]. This is probably the most recognizable term used to describe the effects of being assessed across disciplines [10–12].

Around the same time, an alteration to basic experimental design, the Solomon four-group design, was developed to allow quantification of the size of baseline assessment effects and to control for them [3]. Campbell [13] subsequently proposed that assessments may interact with interventions to either strengthen or weaken observed effects, thus producing biased estimates of effects. The construct of “demand characteristics” [14,15] was subsequently introduced in psychology, referring to the ways in which study participants adjust their responses according to their perceptions of the implicit preferences or expectations of researchers, to be “good subjects” [16].

Four recent systematic reviews summarize and evaluate empirical data on assessment reactivity in brief alcohol intervention trials [7], the Hawthorne effect [17], applications of Solomon four-group designs [18], and demand characteristic studies in nonlaboratory settings [19]. Collectively, these reviews demonstrate that being assessed can impact on behaviors, with small effects usually having been identified, albeit inconsistently, on both self-reported and objectively ascertained outcomes. These are due to being interviewed, completing questionnaires, or being observed. These four reviews do not, however, provide strong evidence of assessment effects as there were substantial weaknesses in the primary studies. Strong and consistent themes to emerge from these studies are the need for a new generation of primary studies dedicated to estimate the size of assessment and other possible research participation effects, and the mechanisms of their production, and the circumstances in which they occur.

## 3 Overlooked prerandomization sources of bias in behavioral intervention trials

The example provided in Box 1 suggests that in such cases, reliable effect estimation has been precluded and thus that randomization has not protected against some form of bias. The reason for this is the violation of a key assumption in conventional trial design and analysis on which the capacity of randomization to prevent bias depends. This is the additivity assumption [20] that the effects of the intervention being evaluated are independent of any possible prerandomization effects of research participation. In simple terms, this implies that it does not matter whether assessment changes behavior or participants react to some other aspect of being researched before randomization because with sufficiently large numbers, randomization guarantees between-group equivalence and ensures that randomized groups differ only in outcomes as a function of the intervention being studied.

Attention has previously been drawn to this additivity assumption in pharmacological trials in mental health [20], although its implications are rarely considered more widely. This assumption is untenable in behavioral intervention trials, most obviously where the research and intervention procedures contain identical content which may affect outcomes. In addition to completing questionnaires, keeping diaries, regular weighing, and using pedometers for both research and intervention purposes, there are other less obvious similarities. For example, making interpersonal declarations of commitment to change, as is often done in providing formal consent, is also a component of many effective behavioral interventions (eg, Ref. [21]). Indeed, self-monitoring and self-regulatory mechanisms through which the research process may influence participants are those same mechanisms that are targeted by behavioral interventions [8,22]. In such scenarios, prerandomization effects cannot be separated from impact on the postrandomization behavior of both intervention and control groups, and biased estimates of effects occur because of this interaction [13,18]. This same issue applies also to drug and placebo effects in pharmacological trials, in which both may confer benefit [20].

Elaborating on Campbell [13], intervention effect estimates could be erroneously diluted when there may be limited capacity for behavior change, which is partially accounted for by prerandomization reactivity, as in the example provided in Box 1. Motivation to change behavior can be thought of as existing on a continuum, with some people more ready to change and others less so [23]. The stimulus provided by research participation may provide sufficient motivation for some people to change their behavior. It is reasonable to suppose that other aspects of the research process could also influence participant cognition, affect, and behavior. This stimulus entails a ceiling effect, and this is likely to be common for preexisting behaviors where participants have thought about the behavior before, have made previous attempts at change, and/or are in a state of contemplation about behavior change [18]. Smoking, sedentary lifestyle, overeating, heavy drinking, or other well-established behaviors about which there are obvious grounds for concern are probably good examples of this situation in which trials may underestimate the true effects of interventions.

We also offer the basis for hypotheses about how intervention effects may be artifactually inflated in trials in which observed effects of the evaluated intervention are contingent on prior preparation provided by the research process. This scenario is most obviously plausible in evaluations of interventions in which reflection on the behavior has been absent previously and is promoted by research participation, thus helping to prepare people for change. This produces a synergistic effect, which may be strongest for the uptake of new behaviors, particularly when some degree of planning for the enactment of the behavior is required [18]. One study included within the Solomon four-group systematic review provided an example of this, where adolescents' completion of a lengthy questionnaire on sexual behavior influenced receptivity to intervention and subsequent condom use outcome [24]. This may be more likely when participants are proactively recruited for intervention on a particular behavior rather than among help seekers or other more active volunteers. It also may be more applicable to health protection rather than existing health-compromising behaviors and more likely in some populations than in others. For example, children and young people may have devoted less time to reflect on some of their behaviors than other populations. It is important to note that the mere existence of any of these interaction effects is sufficient to undermine internal validity because they bias intervention effect estimates.

## 4 Overlooked postrandomization sources of bias in behavioral intervention trials

The problems just described are analogous to the randomization barrier being somewhat porous to the introduction of bias from prerandomization research participation effects. To the extent that the process or outcome of randomization themselves exert direct effects on behavior, these will constitute further sources of bias. Cook and Campbell [25] described the uncertainty inherent in randomization as potentially generating apprehension that can influence outcomes. This is thus another prerandomization source of bias. Reactions to the outcome of randomization, however, are by definition postrandomization. There may be deleterious effects on control group participants when they give up attempts to change, labeled as “resentful demoralization” [25]. Cook and Campbell [25] also used the term “compensatory rivalry” to refer to enhanced efforts at change by securing interventions outside the context of the trial. Such responses are contingent on disappointment at the outcome of randomization. This may occur because it is well established that participants can have preferences for allocation within trials [26], and these preferences can have far reaching consequences, including impacting on trial outcomes [27].

For these reasons, patient preference designs [28] have been developed to avoid randomizing participants with strong allocation preferences to study conditions that would be disappointing. Similarly, Zelen designs [29–31] have also been developed for situations in which seeking consent for randomization may invoke unwanted responses. The use of both designs in many areas beyond the present focus on behavioral intervention trials [32,33] further indicates that these concerns are applicable to experimenting with people in other contexts. We suggest that the underlying nature of the problems posed by expectations and disappointment (apart from in relation to placebo effects [34–36]) and their implications for valid inferences in trials are not widely appreciated, although an article by Colagiuiri [37] is a noteworthy exception. There are valuable qualitative studies illustrating, for example, the dynamic nature of allocation preferences in trials [38], although there are few quantitative studies, other than the patient preference trials themselves. In our perspective, preferences arise out of the interaction of the participant and the research process. Participants may bring not only allocation preferences but also a wide array of hopes and concerns, motivations and uncertainties, and other cognitions and emotions to their involvement in trials that are more or less intrinsic to experimenting with people. This situation calls for careful deliberation in study design and vigilance for the intrusion of significant biases arising from these interactions.

Another possible source of bias after randomization to which little attention has previously been given is when there are seemingly trivial differences in the follow-up assessments completed by each group. This situation could arise, for example, when the intervention group is required to provide feedback data on the intervention. This could cause participants to reflect on their behavior differentially between groups and introduce bias to subsequent follow-up assessments. This constitutes an example that fits well with current thinking about performance bias [39,40], where seemingly minor differences in research conditions contribute to different outcomes. This construct is useful because it directs attention to what is done to or with study participants. In addition to these examples we have provided, other postrandomization sources of bias such as those associated with compliance to allocated interventions are much better understood, and consequently, there are analytical strategies developed for dealing with them [41,42]. For postrandomization sources of bias, simple main effects on later outcomes and more complex interaction effects introduce bias.

Even when there are no differences at all in the content of follow-up data collection or other postrandomization study procedures, the intervention group being reminded of intervention content, and it being thus reinforced, can be a means by which bias is introduced. This situation is not well captured by the existing definition of performance bias as the bias originates not in any differences in how participants are treated by the research study [39] but in how participants respond differently, specifically as a result of their allocation. It is a moot point how well the construct of performance bias captures this type of problem.

## 5 The need for new research on these sources of bias

We propose that hypotheses concerning the main effects of prerandomization artifacts warrant testing in two-arm experimental studies as precursors to more complex evaluations of interaction effects in four-arm studies. For example, if assessments do not have main effects, their possible interactions with randomization outcomes are much less promising targets for investigation. For two-arm experiments, manipulations of the process of informed consent, for example, knowing that randomization will occur or what is the particular behavior under investigation [43], provide examples of possible targets for study.

Interactions between recruitment or assessment effects and the outcome of randomization produce bias in behavioral intervention trials, as do the main effects of postrandomization artifacts such as responses to either earlier follow-up assessments or randomization outcome other than precisely as intended by the design of the intervention–control contrast [44]. The basic design structure of the tests of these hypotheses is straightforward. In the four-arm trial in the manner of the factorial or Solomon four-group design, which randomizes to intervention or assessment exposure, both or neither, content does not need to be restricted to investigation of assessment effects. For example, individual informed consent could be experimentally manipulated in this way, with the procedure either omitted or altered in a study in which intervention exposure is also randomized. The ethical challenges involved are arguably more complex than the study design considerations, and we have elaborated elsewhere justifications for the use of deception in relation to both methodological and substantive evaluation studies of brief alcohol intervention effectiveness [45].

We suggest that specifying the optimal research conditions in which these types of studies should be undertaken is more difficult to do, however, than producing the basic structure of the study designs. Which behaviors, populations, settings, and interventions are most promising for investigation? Some suggestions have been made here. Eliciting the experiences and views of researchers [46] and securing their collaboration in doing this research will help identify priorities for such studies, as will facilitating direct contributions from research participants themselves.

Research participation effects may be implicated in other more well-known threats to valid inference [40]. They may interact with attrition bias to produce differential follow-up rates between study groups. Similarly, if participants differentially underreport risk behaviors because of their allocation, this can lead to detection bias. Behavioral intervention trials often necessarily rely on self-reported behavioral data, and such studies may be especially vulnerable to these effects [47]. In both examples provided here, detailed scrutiny of the literature on these better known forms of biases [48] may be useful to understand the potential for the types of research participation effects considered here to produce bias. Although blinding may be used to protect against various forms of bias, it may be less available and thus less useful in behavioral intervention trials than elsewhere [49].

History demonstrates disappointingly slow progress in thinking about the nature of the problems described here and in successfully studying them [19]. A conceptual framework (eg, Ref. [50]) will need to be built over time, probably informed by both qualitative and quantitative data, and evaluation of the difficult ethical issues involved in deception can be informed by dedicated methodological studies (see Ref. [51]). Further work on this subject may well require the development of new terminology to overcome existing disciplinary and research topic barriers. Existing constructs such as the Hawthorne effect and demand characteristics lack specificity and permit too many meanings to be useful when used alone.

Notwithstanding these challenges, the authors contend that the need for this research is no longer ignorable in relation to behavioral intervention trials. It is not clear how important attention to these issues is in other types of trials, and this question merits consideration. Behavioral intervention trials that are sensitive to the issues raised here may have interesting design characteristics. The AMADEUS-1 trial, for example, blinded all participants to involvement in the trial at all stages of the study and used a no-contact control group for comparison with intervention groups in receipt of routine practice [52]. This is an unusual example of an unobtrusive evaluation of service provision specifically designed to avoid or minimize research participation effects. Trial outcomes further demonstrated the substance of these concerns, where assessment-only had very similar effects to assessment and feedback compared with no contact [53]. Preliminary suggestions applicable to the design and conduct of more conventional trials are offered in Box 2.

## 6 Conclusions

There has been no attempt here to produce a fully comprehensive guide to possible research participation effects in behavioral intervention trials. For example, we should expect that reasons for participation in these types of studies will be important to the existence and the nature of any research participation effects [54]. Routine practices such as paying people to participate should be expected to have some impact on both these reasons for participation and possibly also their subsequent relationship with the research [55]. We suggest that the construct of research participation effects can provide a useful basis for more comprehensive evaluations of the possible problems discussed here.

There are also obvious solutions to some of these possible problems, by omitting when possible the aspect of study conduct believed to be responsible. These problems are very likely to be widely amenable to elimination by design, or statistical control if not, as they are artifacts of the decisions made by researchers. When a possible source of such bias is identified and it is not clear how much of a threat to validity may be entailed, the likely benefit of the data gained with this design decision must be considered in relation to the risk of bias. This pragmatic cost—benefit appraisal may resemble how research decisions are routinely made.

The thinking presented here on possible problems arising from experimenting with people in behavioral intervention and other types of trials does not provide reasons to abandon them. As Hollon [56] has remarked “to paraphrase Churchill on democracy, RCTs are fallible and far from perfect; the only good thing that we can say about them is that they are better than the alternatives.” We suggest that attention to possible research participation effects provides a means by which RCTs can be improved in delivering less biased estimates of behavioral and other intervention effects, if and when this is needed.

## Box 1 An hypothetical example using smoking cessation data [57]

The most effective smoking cessation behavioral interventions such as high-intensity counseling result in a true cessation rate of approximately 22%. Recruitment and assessment in trials can provide a stimulus to quit, and reinforcement of this decision, for those who are most ready, willing, and able to successfully change this behavior. This yields an approximately 11% cessation rate in control conditions in these same studies. This exceeds both the cessation rates of approximately 3% and 6% seen in unscreened and screened smokers, respectively. In an RCT, only a further 11% of those allocated to intervention will be responding to the evaluated intervention itself. In this situation, outcomes in the trial will be 22% for the intervention group compared with 11% for the control group, an 11% difference, which is a biased estimate of the true effect.

## Box 2 Trial design considerations

1. Incorporate examination of potential for research participation effects in pilot investigations of all trial procedures in which there may be any concern.
2. Ask participants whether and how research participation affects them in formal qualitative and quantitative studies nested within trials.
3. Collect and analyze data on routine and seemingly unremarkable aspects of the research process, on both formal and informal contacts.
4. Minimize interpersonal contacts and be as unobtrusive as possible.
5. Be careful with research assessments obtained directly from participants.
6. Consider the possible benefits of baseline data collection in relation to the possible risk of bias.
7. Undertake randomized substudies within trials to measure potential research artifacts.
8. Explore all available content options for blinding.
9. Evaluate the use of blinding and deception, from both ethical and methodological perspectives.
10. Ensure no aspects of trial design and conduct interfere with the precise experimental contrast that answering your research question demands.
